# Skin cancer knowledge and attitudes in the region of Fez, Morocco: a cross-sectional study

**DOI:** 10.1186/s12895-017-0055-8

**Published:** 2017-02-17

**Authors:** Awatef kelati, Hanane Baybay, Mariam Atassi, Samira Elfakir, Salim Gallouj, Mariame Meziane, Fatima Zahra Mernissi

**Affiliations:** 1 Department of dermatology, University Hospital Hassan II, 202 Hay Mohamadi, Fez, Morocco; 2 Department of clinical epidemiology and scientific research, University Hospital Hassan II, Fez, Morocco

**Keywords:** Cross-Sectional study, Skin cancer, Epidemiology, Knowledge, Attitudes, Morocco

## Abstract

**Background:**

The prevalence of skin cancers is constantly increasing in Morocco, and they have gradually become more aggressive due to a significant delay in the diagnosis. Our aim was to assess the levels of awareness and the influencing factors related to skin cancer knowledge in Morocco.

**Methods:**

This cross-sectional study was carried out in Morocco through the medium of a validated questionnaire, which contained several items – demographics, skin cancer knowledge and attitudes towards skin cancer patients– during a period of 1 year (2014).

**Results:**

Out of the 700 participants enrolled in the study, 17.9% had never heard of skin cancer, 32.5% had a low score of skin cancer knowledge, 66.7% had a moderate score, and only 0.85% had a high score of skin cancer knowledge. Further, 15.1% of the participants were under the assumption that this cancer is contagious. The sun was the most incriminated risk factor in skin cancer occurrence by 74.3% of the participants, and 57.9% of them believed that prevention is important through using various means of photoprotection. After univariate and multivariate analysis, the influencing factors related to the skin cancer knowledge in Morocco were: the socioeconomic status (*P* = 0.003*,* OR = 7. 3) and the educational level (*p* < 0.001*,* OR = 20. 9).

**Conclusions:**

Due to the lack of knowledge or the underestimation of skin cancer in our study population, efforts are needed to promote skin cancer surveillance behaviors in Morocco.

## Background

Skin cancer (SC) is the most common worldwide malignancy and it a preeminent global public health problem [1]. These SCs are divided into two main groups in Morocco: melanoma and non-melanoma SCs (NMSC), these last tumors are mainly basal cell carcinoma (BCC) and squamous cell carcinoma (SCC), and also cutaneous lymphomas, Sarcomas in addition to rare types such as adnexal tumors and Merkel cell carcinoma [2].

In Morocco, because of the absence of a national cancer registry, the exact number of incidence and mortality of SC is not available. However, according to regional records -Casablanca register of cancers (2004), Cancer registry of the National Institute of Oncology Sidi Mohamed Ben Abdellah, Rabat (2002–2007) and Rabat register of cancers (2009)- and a few publications, a change in the distribution of different SCs was noted; the prevalence of melanoma and lymphoma increased from 3,5% and 1,5% to 10,4% and 18,6% of skin malignancies in a period of 19 years; while the prevalence of the SCC and BCC decreased from 58% and 32% to 26,9% and 23,7% in the same period [2]; with a decrease in the proportion of skin carcinomas at the expense of Melanoma: 50.6% of carcinomas during the period 1992–2011 against 90% during the period 1971–1991.

There were also an increase in melanomas’ aggressivity, in a study of 30 melanomas made in the region of Fez-Boulmane [3], Breslow index was more than 4 mm in 33%, 56 patients had metastasis and 2 patients died; while these numbers have almost doubled in a recent cohort of 70 cases of melanoma, carried out in the same region (unpublished data of the Moroccan Society of Dermatology), the Breslow at the moment of the diagnosis was > 4 mm in 50% of cases, with 4 cases of death.

Because of this increase, the costs attributable to the diagnostic delay and noneffective health care procedures are now an economic burden and they are a problem for health care services worldwide, for example, Medical costs to treat SCs in the USA are estimated at $3 billion annually [4].

However, the most important fact about SC is that it is mostly preventable with the health care promotion and the early detection endeavors. [5] When discovered early, the survival rate for individuals with melanoma is > 98%, as compared with 15% of those diagnosed with advanced disease. That’s why health care providers should be promoting the establishment of appropriate strategies to ultimately improve the preventive provision of care for these cancers, by carrying out programs to evaluate the degree of awareness of populations with this problem and the benefit of the prevention of these risk factors especially sun exposure. Kyle and al. [5] reported that every dollar spent on sun safety educational initiatives saves the nation almost $4 in health care costs, in addition to a reduction in morbidity and mortality associated with SC.

In emerging Countries like Morocco, SC is not a public health priority, and it is usually underestimated. For this reason, before thinking about effective preventive measures, studies must be carried out to investigate SC knowledge in our populations.

The aim of the present study was to assess the level of awareness, and the influencing factors related to SC knowledge in Morocco, and to detect attitudes towards SC patients based on the degree of awareness of our population.

## Methods

### Study design

This was a cross-sectional study, spread over a period of 1 year between March 2014-March 2015 in the city of Fez in Morocco.

### Participants

Participants aged more than 18 years old were randomly selected from different categories of the Moroccan population with different educational levels (ELs) and socioeconomic levels using a systematic sampling method: from the daily list of patients of other specialities’ consultations and their accompanists in the Hospital Hassan II of Fez, Morocco.

### Data collection

The data collection was based on filling a five-minute questionnaire by participants, in a “face to face” way with the investigator, in order to help them especially those with a low EL who can’t read the questionnaire by themselves. Investigators were volunteers from the medical staff of the departments of dermatology and clinical epidemiology of the Hospital Hassan II of Fez.

The questionnaire consisted of 42 questions with several items: demographics, SC knowledge, attitudes towards SC patients, use of photoprotection measures, and the relationship with the doctor. The questions about SC knowledge were included in Table 1.

**Table 1:** The questions about SC knowledge

Questions of SC knowledge	
1) Have you ever/never heard of skin cancer? 2) Is it dangerous? 3) Could It kill 4) Clinical manifestations (multiples choices) (Table 3) 5) Alarming symptoms (multiples choices) (Table 3) 6) Skin cancer risk factors (multiples choices) (Table 3) 7) Its relationship with the phototype 8) Do you think that it appears in a pre-existing lesion? 9) Is mucosal involvement possible? 10) Do you know someone who has this skin cancer? 11) The reaction of the participant towards skin cancer patient 12) Relationship with the doctor: how should the doctor announce the diagnosis?	

This questionnaire was validated in a multidisciplinary meeting, including experts in Dermatology, Clinical epidemiology, Scientific research and a psychiatrist.

Epidemiological and sociodemographic data of participants included: age, gender, highest level of education, health insurance status, profession and salary, medical records of participants and their phototype (filled by the investigator); then, participants were asked if they heard about SC and from who (health professional, relatives or media) and if they know someone who suffers from this disease and how he lived the experience; then, they completed questions regarding their knowledge about SC (risk factors especially sun exposure, clinical manifestations, location, contagiosity and treatment), in addition to SC eventually preventive measures (sunscreen use, shade seeking, and use of sun protective clothing).

Also, the questionnaire explored the participant’s opinion of how must be the relationship between the physician and the patient, and how to announce the diagnosis (direct way or progressive way), and if the psychiatric care is obligatory for patients instead of, or accompanied by the family support.

Participants used a 3-point response scale (yes, no, I don’t know or others) to indicate their response to the questions.

A score of SC knowledge level was established based on correct answers from 0 to 27: a low level of knowledge was described if the participant had less than 10 correct answers, a moderate level of knowledge if the participant had between 10 and 20 correct answers, and a high level of knowledge if the participant had more than 20 correct answers.

Socioeconomic level (SEL) was considered low if the monthly salary was less than 3000 DH (294.30 USD), moderate if it was between 2000DH (196.20 USD) and 7000DH (686.70 USD), and high if it was more than 7000 DH (686,70 USD).

### Statistical analysis

A descriptive, univariate and multivariate analysis using the SPSS 20 software were performed.

In the descriptive analysis, quantitative variables were expressed by means ± standard deviation and qualitative variables by percentages. In the univariate analysis, the “Chi-square” test was used to compare percentages in order to determine the factors associated with the knowledge level. In the multivariate analysis, high and moderate levels of knowledge were grouped into one group of an appropriate level of SC knowledge, and a logistic binary regression was performed including variables for which the p value in the univariate analysis was less than 0.20, then a step down method was carried out. A p value less than 0.05 was considered statistically significant.

## Results

We had included 700 subjects in this survey, the average age of participants was 33.6 years (SD = 13. 3 years). There was a female predominance (61.9%) and 64.1% of participants were in a moderate SEL (Table 2).

**Table 2:** Descriptive analysis of epidemiological characteristics of participants

Epidemiological characteristics	*N* = 700	%
Age groups		
< 15 years old	5	0.7
15–45 years old	548	78.3
> 45 years old	147	21.1
Gender		
F	438	62.5
M	262	37.4
Phototype		
III	93	13.2
IV	489	69.8
V	118	16.8
Educational level		
Academic (university)	357	51
High school	164	23.4
Primary education	68	9.7
Illiterate	111	15.9
Socioeconomic level (SEL)		
Low SEL	167	23.8
High SEL	68	9.7
Moderate SEL	449	64.1

17.9% of the participants had never heard of SC, and 15.1% of them thought that it was contagious.

32,5% of the participants had a low score of SC knowledge level, 66.7% had a moderate score and only 0.85% had a high score of SC knowledge level (Table 3).

**Table 3:** Descriptive analysis of the questionnaire items about skin cancer knowledge

Items	*N* = 700	%	Influencing factors	*p* value
Have you ever heard of skin cancer?				
Yes	486	69.7	None	
No	125	17.9		
I don’t know	86	12.3		
Sources of Information			Age	0.03
Internet	350	50	Educational level	0.02
TV	290	41.4		
Patients and doctors	60	8.6		
Is it dangerous?			None	
Yes	384	54.8		
No	70	10		
I don’t know	246	35.1		
It could kill				
Yes	304	44.3	None	
No	250	35.7		
I don’t know	146	20		
Clinical manifestations			None	
Papules	234	54.3		
Nodules	126	30.1		
Tumors	174	41.5		
Ulcers	213	50.8		
Purulent bubbles	121	28.9		
Black patches	20	2.9		
Others	384	54.8		
Alarming symptoms			None	
Size increase	149	31.7		
Color change	143	30.4		
Resistance to usual treatment	118	25.2		
Pruritus	39	5.6		
Skin cancer risk factors			None	
Sun Exposure	344	74.3		
Genetic factors (Family history of skin cancer)	235	51.1		
Chemical products	230	49.8		
Irradiation	207	44.7		
Infection	171	37		
Smoking	121	26.5		
Relationship with the phototype (324/46.3%)			None	
Fair skin	300	42.8		
Dark skin	70	10		
Appears without pre existing lesion	142	20.2	None	
Appears in pre existing lesion	331	47.2		
Mucosal involvement	312	44.5	None	
Treatments			None	
Surgery	174	24.8		
Chemotherapy	223	31.8		
Radiotherapy	194	27.7		
Total Score				
Low level of knowledge	228	32.5		
Moderate level of Knowledge	467	66.7	Socioeconomic level	<0.001
High level of knowledge	6	0.85	Educational level	<0.001

The sun was the factor the most incriminated in the pathogenesis of SC (74.3%) and 57.9% of the participants believed that it is important to use various means of photoprotection as sunscreens (34.2%), clothes (11%) or avoiding the exposition to the sun between 10 am and 16 pm (29%).

Regarding the relationship between the doctor and the patient, 58.4% of the participants thought that the doctor has to announce the diagnosis of cancer to the patient gradually, while 23.3% of the participants preferred the direct way, and 18.3% of them thought that the diagnosis must be hidden to the patient (Table 4).

**Table 4:** Descriptive analysis of participants' responses about behaviors towards skin cancer patients and the relationship with the doctor

Items	*N* (%) = 700	Men *N*(%) 262 (37.4%)	Women *N*(%) 438(62.5%)	Influencing factors	*p* value
Relationship with skin cancer patients					
Do you know someone who has this skin cancer?				None	
Yes	139 (19.9)	55(21)	84 (19.2)		
No	561(80.1)	207(79)	354 (80.1)		
The reaction of the participant towards skin cancer patient				Educational level	<0.001
Remoteness (contagious)	90 (15.1)	34 (13)	56 (12.7)		
Indifferent	181 (30.4)	75 (28.6)	106 (24.2)		
Support	324 (54.5)	153 (58.4)	276 (63)		
The psychiatric care is obligatory for skin cancer patients				None	
Yes	358 (51.1)	122 (46.5)	233 (53.1)		
No	202 (29)	33 (12.6)	40 (9.1)		
I don’t know	140 (20)	107 (40.8)	165 (37.6)		
Relationship with the doctor: how should the doctor announce the diagnosis?				Gender	0.02
Direct way	157 (23.3)	99 (38)	84 (19.1)		
Progressive way	393 (58.4)	147 (56.1)	265 (60.5)		
Hiding the diagnosis to the patient and inform just his family	123 (18.4)	16 (6.1%)	89 (20.3)		

In the univariate analysis, younger participants (<45 years) and subjects with a moderate or a high SEL (*p* < 10–4) and a high EL (*p* < 0.001) had an appropriate level of SC knowledge, and they emphasized the importance of the photoprotection and the psychiatric care for these patients.

Sources of information about SC varied according to the age (*P* = 0. 03) and the EL (*P* = 0. 02). Each category of the population had a different source of information; young persons with a moderate and a high EL used the internet, while television was the source of information of illiterates and aged persons.

Behaviors of the participants were influenced by their EL; the more the EL was increased, the more the participants preferred to stay away from SC patients or they remain indifferent to them because it could be contagious, and it was analphabets and the persons with a low EL who thought that they must support these patients even though it could be contagious (*p* < 0.001).

Men preferred the direct relationship between the doctor and the patient, especially the way to announce the diagnosis of cancer (38%), while women thought that the doctor must announce it gradually (60.5%) or not to announce it at all (20.3%) (*p* = 0.02) (Table 4).

After the multivariate analysis, the influencing factors related to SC knowledge in our Moroccan population were the SEL (*P* = 0. 003, OR = 7. 306, IC 95% = (1.9–27.5)), and the EL (*p* < 0.001, OR = 20.9, IC 95% = (10.5–41.6)) (Table 5).

**Table 5:** Multivariate analysis showing the association between factors and SC knowledge score

Variables	*P* value	OR	CI 95%	
SEL				
Low SEL	0.000	4.232	2.550	7.023
Moderate and high SEL	0.003	7.306	1.936	27.567
Educationnal level				
Academic (university)	0.000	20.996	10.584	41.651
High school	0.000	3.332	1.834	6.052
Illiterates and primary education	0.038	2.136	1.041	4.382

## Discussion

SC is the most common malignancy [6–14]; and it increases dramatically, especially Melanoma [2], which has a faster growing incidence and a higher mortality rate than that of any other malignancy [15]. The underlying reasons for this increase were discussed in many studies that have confirmed the development of risk factors such as sun exposure; chronic and repeated in both BCC and SCC, while intense sun exposure and a history of sunburn was linked to melanoma [3]. Furthermore, a history of multiple moles (>50), atypical moles, light skin, red or blond hair, blue or green eyes, freckles, history of sunburn, or family history of melanoma are considered higher risks for developing melanoma [4], other risk factors especially for NMSC are an immunosuppression, human papilloma virus, chronic wounds and burns…

Compared with other cancer types, SC treatment costs are currently low because it can be primarily treated efficiently in an office-based setting. NMSC care cost stands in fifth place after prostate, lung, colon, and breast carcinomas. But, due to its considerable frequency, its final cost would be huge and depends on 2 factors: care settings and treatment modalities [2–16].

This present study is -to our knowledge- the first Moroccan, North African and among the first occidental studies interested in assessing the knowledge level, attitudes, and behaviors related to SC. Apart from three similar studies conducted on youths in Maryland [17] and in the USA [18], and the survey of Gordon R and al. that concluded to an overall low levels of knowledge about SCs especially melanoma [19, 20]. Other few studies interested only in the relationship between the sun, the photoprotection and SCs were performed [21–26], or conducted on minorities and targeted populations like golfers [6]. Similarly to these studies, the sun was the factor the most incriminated in the etiopathogenesis of skin cancer in our study (74.3%), and 57.9% of the participants believed that it is important for the SC prevention using various measures especially sunscreens.

We have found that 17.9% of participants, especially those with a low EL had never heard of SC, which is a real issue because this category not only underestimated this cancer and may have a poor sense of risk-reducing strategies for SC, but they would never come to see the doctor in the early stage of the disease, which explain why we still see historical tumors with bad prognosis and metastasis. Also, another possible factor of delay in our context is the frequent use of traditional medicine such as fire and plants.

Another common error in our population is the idea that SC is contagious, which was the participants’ opinion in 15.1% of cases; and that it is due to germs (37%). This idea of contagious disease would not only influence the patient, but also his integrity in the society, which will surely make the situation more complicated with a real impact on the patient’s life quality.

What was surprising in our results was the fact that the more the EL increased, the more the participant preferred to stay away from SC patients or remain indifferent to them. While persons with a low EL thought that they must support these patients even though it could be contagious, this could be explained by the importance of the family and the religious links in our population, particularly in those with a low and moderate SEL who share a common home and a lifestyle.

From another perspective, 69.7% of our participants, especially younger patients with moderate or high SEL and EL had already heard of this type of cancer from different sources, such as SC patients, dermatologists and particularly the media (TV, internet..). This media tools may be an interesting way of sensitization that we can use as a strategy of SC prevention. According to our results, each category of the population may be educated using different media tools; for young educated persons the internet and the social media are the most effective way of giving information, while TV remains the best way of sensitization for illiterates and aged persons.

Regarding the way of announcing the diagnosis of cancer to the patient, Our results confirmed that it must vary according to the gender. This fact must be taken into account by the health care professionals in Morocco in order to decrease the disease burden, especially in women, but in the same time, we should be careful, because, it may lead the patient to neglect his disease if he did not know the gravity of it.

Based on these results, we deduced that it’s crucial to seek ways to correct the false ideas and the common errors regarding this special type of cancer in an attempt to prevent and to establish a SC surveillance and to facilitate its early detection.

Unfortunately, Guidelines regarding the SC screening are inconsistent and depend on the economic level of countries and natural factors (sunshine, climate, altitude), phototype and customs. However, several prominent national organizations recommended and emphasized sun-protective behaviors [27], education of health professionals, including nurses, in addition to improving strategies of SC and melanoma screening [28–32].

Ultimately, Despite all the barriers to SC prevention in Morocco, like financial issues, the lack of national guidelines, awareness, and availability of physicians. This overall burden must lead the health system in Morocco to consider this serious health problem and to try to establish a general approach to deal with it, using different ways of communication like the internet [33]; the TV and the programs of teaching health maintenance, the school programs for children; the sun protection guidelines and the SC screening programs not only for dermatologists, but for all the health care professionals.

This study is not without limitations, especially concerning our choice of participants; all the sections of the population were represented in the study sample, but the percentage of these sections was not equal. Although participants were chosen randomly and we tried to have a representative sample of all categories of the population, 78.3% of our participants were young (between 18 and 45 years old) and having an academic degree in 51% of cases.

## Conclusion

Due to the lack of knowledge or the underestimation of SCs in our population, as we proved it in our study, efforts are needed to promote SC’s surveillance and screening in Morocco, in addition to the establishment of effective campaigns of SC sensitization.

## Abbreviations

BCC: Basal cell carcinoma
EL: Educational level
NMSC: Non-melanoma skin cancers
SC: Skin cancer
SCC: Squamous cell carcinoma
SEL: Socio economic level
